# Cancer cell proliferation is inhibited by specific modulation frequencies

**DOI:** 10.1038/bjc.2011.523

**Published:** 2011-12-01

**Authors:** J W Zimmerman, M J Pennison, I Brezovich, N Yi, C T Yang, R Ramaker, D Absher, R M Myers, N Kuster, F P Costa, A Barbault, B Pasche

**Affiliations:** 1 Division of Hematology/Oncology, Department of Medicine, University of Alabama at Birmingham and UAB Comprehensive Cancer Center, 1802 6th Avenue South, NP 2566, Birmingham, AL 35294-3300, USA; 2 Department of Radiation Oncology, University of Alabama at Birmingham and UAB Comprehensive Cancer Center, Birmingham, AL 35294, USA; 3 Section of Statistical Genetics, Department of Biostatistics, School of Public Health, The University of Alabama at Birmingham, Birmingham, AL 35294, USA; 4 HudsonAlpha Institute for Biotechnology, Huntsville, AL 35806, USA; 5 IT'IS Foundation, Swiss Federal Institute of Technology, Zurich, Switzerland; 6 Department of Transplantation and Liver Surgery, Hospital das Clínicas, University of São Paulo, São Paulo, Brazil; 7 Rue de Verdun 20, Colmar 68000, France

**Keywords:** hepatocelullar carcinoma, electromagnetic fields, mitotic spindle, PLP2, XCL2

## Abstract

**Background:**

There is clinical evidence that very low and safe levels of amplitude-modulated electromagnetic fields administered via an intrabuccal spoon-shaped probe may elicit therapeutic responses in patients with cancer. However, there is no known mechanism explaining the anti-proliferative effect of very low intensity electromagnetic fields.

**Methods:**

To understand the mechanism of this novel approach, hepatocellular carcinoma (HCC) cells were exposed to 27.12 MHz radiofrequency electromagnetic fields using *in vitro* exposure systems designed to replicate *in vivo* conditions. Cancer cells were exposed to tumour-specific modulation frequencies, previously identified by biofeedback methods in patients with a diagnosis of cancer. Control modulation frequencies consisted of randomly chosen modulation frequencies within the same 100 Hz–21 kHz range as cancer-specific frequencies.

**Results:**

The growth of HCC and breast cancer cells was significantly decreased by HCC-specific and breast cancer-specific modulation frequencies, respectively. However, the same frequencies did not affect proliferation of nonmalignant hepatocytes or breast epithelial cells. Inhibition of HCC cell proliferation was associated with downregulation of *XCL2* and *PLP2*. Furthermore, HCC-specific modulation frequencies disrupted the mitotic spindle.

**Conclusion:**

These findings uncover a novel mechanism controlling the growth of cancer cells at specific modulation frequencies without affecting normal tissues, which may have broad implications in oncology.

Treatment of hepatocellular carcinoma (HCC) is a major challenge given the limited number of therapeutic options available (Thomas and Zhu, 2005). We have developed a novel approach to treat advanced HCC, consisting of intrabuccal administration of very low levels of radiofrequency electromagnetic fields (RF EMF), amplitude-modulated at specific frequencies, and identified using biofeedback methods in patients with cancer (Barbault *et al*, 2009). The encouraging findings from a feasibility study (Barbault *et al*, 2009) led to the design of a phase I/II trial in patients with advanced HCC, and objective responses assessed by CT-scan and changes in alpha-fetoprotein levels were observed in several patients with biopsy-proven HCC (Costa *et al*, 2011). These findings prompted us to initiate reverse translational experiments to investigate the mechanism of action of amplitude-modulated electromagnetic fields. Two different *in vitro* exposure systems operating at 27.12 MHz were used to expose cells in culture, replicating patient-treatment conditions.

Proliferation of both HepG2 and Huh7 HCC cells was significantly decreased upon exposure to radiofrequency electromagnetic fields, which were modulated at HCC-specific modulation frequencies. To determine how such frequencies modulate cancer cell growth, we assessed differential gene expression with RNA-seq and found that the expression of several genes was significantly downregulated by HCC-specific modulation frequencies. Previous reports have shown that low intensity, intermediate frequency electric fields are capable of inhibiting cancer growth by interfering with the proper formation of the mitotic spindle (Kirson *et al*, 2004; Kirson *et al*, 2007). Similarly, we found that electromagnetic fields that are amplitude-modulated at HCC-specific frequencies disrupt the mitotic spindle of HCC cells. Thus, we provide novel evidence that very low level of amplitude-modulated electromagnetic fields block the growth of HCC cells in a tumour- and tissue-specific fashion.

## Materials and methods

### *In vitro* exposure devices

**Figure 1:**
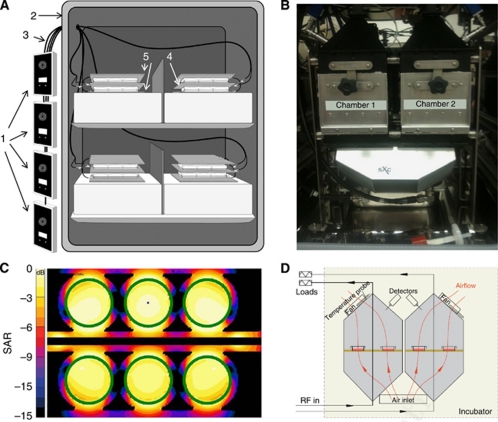
*In vitro* exposure experimental setups. (**A**) Parallel plate capacitor. Emitting devices (1) are placed outside the incubator (2). Each device is connected to a coaxial cable (3), which is connected to a set of brass plates inside the incubator. The centre brass plate (4) is connected to the inner conductor of the emitting device coaxial cable. The outer two brass plates (5) are connected to the outer conductor of the emitting device coaxial cable. Plates containing cells are placed in between the brass plates. (**B**) TEM cell. The system contains two identical TEM cells placed in an incubator. (**C**) Distribution of the specific absorption rate (SAR) of cell monolayer in the TEM cell (1 dB per contour), (**D**) Schematic representation showing the air flow through the TEM cell.

The design and construction of the two *in vivo* exposure devices (Figure 1) used to conduct these experiments is described in the Supplementary Information.

### Cell lines

HepG2 and Huh7 cells, both of Biosafety Level 1, were used as representative HCC cell lines. HepG2 cells were obtained from ATCC (Manassas, VA, USA), and Huh7 cells were a gift from Dr Nareej Saxena (Emory University). Normal hepatocytes, THLE-2 cells, were also obtained from ATCC. The breast adenocarcinoma cell line MCF-7 was used as a representative non-HCC malignant cell line (ATCC). The breast epithelial cell line MCF-10A (ATCC) was used to represent normal breast cells. Lymphoblastoid cell lines from healthy individuals enrolled in IRB-approved protocols were provided by Dr Jeff Edberg (UAB).

### [^3^H]thymidine incorporation assay

Growth inhibition (GI) was assessed in HCC cells exposed to HCC-specific modulation frequencies as previously described (Rosman *et al*, 2008).

### Luminescent cell viability assay

Cell proliferation was quantitated using the Promega CellTiter-Glo Luminescent Cell Viability Assay (Promega, Madison, WI, USA), a method to determine the number of viable cells in culture based on ATP quantitation.

### RNA-seq

We performed RNA-seq as previously described (Reddy *et al*, 2009). We used HepG2 cells exposed to either HCC-specific modulation frequencies or to randomly chosen frequencies. We double-selected polyA-containing mRNA from 3 *μ*g of total RNA by using oligo-dT magnetic beads. We fragmented the mRNA with RNA fragmentation buffer and removed free-ions with a G-50 Sepharose spin column. Fragmented mRNA was used as a template to synthesise single-stranded cDNA with SuperScript II reverse transcriptase with random hexamer primers in the presence of RNAseOUT (Invitrogen by Life Technologies Corporation, Carlsbad, CA, USA). We synthesised double-stranded DNA (dsDNA) for sequencing by ligating Illumina (Illumina, San Diego, CA, USA) sequencing adaptors to blunted and dA-extended dsDNA, and size-selected fragments of 200–300 bp from a 2% Invitrogen gel and purified with a Qiagen Gel Extraction kit (Qiagen, Valencia, CA, USA). Lastly, we amplified the dsDNA library with 15 rounds of PCR with Illumina sequencing primers. Sequencing was performed on an Illumina GenomeAnalyzer IIx and the paired 36 bp reads were mapped to the hg18 reference genome by using ELAND (Illumina), allowing up to two mismatches per read and 10 or fewer map locations. By using the ERANGE software package (http://woldlab.caltech.edu/rnaseq), we placed uniquely mapped reads against 29 673 transcripts from NCBI build 36.1 of the human genome. After placing unique reads, ERANGE assigned multiple mapping reads and reads mapping to splice junctions according to the number of unique reads in potential transcripts. Once all reads were mapped, ERANGE reported gene expression in units of reads per kilobase of exon and per million tags sequenced (RPKM).

### Quantitative PCR

At the conclusion of the AM-EMF exposure portion of the experiment, RNA extraction (Qiagen) and reverse transcription (TaqMan, Applied Biosystems by Life Technologies Corporation) were performed to generate cDNA. Experiments comparing gene expression in HCC cells receiving HCC-specific AM-EMF with gene expression in HCC cells not receiving any exposure were conducted using Applied Biosystems pre-designed TaqMan Gene Expression Assays (*PLP2,* cat#Hs01099969_g1; *XCL2,* cat#Hs00237019_m1; Applied Biosystems by Life Technologies Corporation). Real-time quantitation was completed in quadruplicate according to the manufacturer's instructions using an ABI 7900HT Real-Time PCR System (ABI by Life Technologies Corporation), with analysis performed using ABI SDS2.2 software. Quantitative values of gene expression were determined by comparing PCR amplification curves to a known standard curve generated in tandem with the experimental samples. Each sample was individually normalised to the average corresponding to endogenous expression of *GAPDH* (*GAPDH*, cat#Hs99999905_m1, TaqMan, Applied Biosystems by Life Technologies Corporation). Averages of the normalised values from each condition were then used to compare the relative gene expression between the experimental groups. The s.e.m. was determined for each experimental condition.

### Confocal laser scanning microscopy

Cells undergoing mitosis were imaged using the Zeiss LSM 710 Confocal Laser Scanning Microscope (Carl Zeiss, Inc., Thornwood, NY, USA). For imaging experiments, 22 mm square microscope cover glass (Corning Life Sciences, Lowell, MA, USA, cat#2865–22) were flame-sterilised with 200-proof ethanol and placed in 6-well or 35 mm Falcon tissue culture plates (BD Biosciences, Franklin Lakes, NJ, USA). Approximately 300 *μ*l of cell suspension/growth media was added directly to the top of the cover slips, and cells were plated at varying concentrations (4 × 10^5^–5 × 10^5^ cells per ml) on separate cover slips for each assay to control for variability in antibody affinity between different experiments. Once the cells were given 8–18 h to attach to the cover slips, 3 ml of complete growth media was added to each well containing a cover slip. Following RF EMF exposure, indirect immunofluorescent microscopy compared the cells receiving HCC-specific modulation frequencies with cells not receiving any exposure (Microtubule Marker (AE-8) sc-73551, Fluorescent Secondary Alexa Fluor 488 goat anti-mouse IgG (H+L): A-11001; Santa Cruz Biotechnologies, Santa Cruz, CA, USA).

### Karyotype analysis

To determine whether these changes were associated with karyotypic changes, HepG2 cells exposed to HCC-specific modulation frequencies or unexposed were harvested, slides prepared, and metaphase chromosomes G-banded using standard methods. The chromosomes were analysed and the karyotype described according to the International System for Cytogenetic Nomenclature (Brothman *et al*, 2009).

### Statistical analyses

One sample two-sided *t*-test was performed to test the significance of cell proliferation exposed to RF EMF amplitude-modulated at tumour-specific or randomly chosen frequencies. ANCOVA analysis: For the long-term (7 weeks) GI analysis and the GI analysis for varying SAR values (0.05, 0.1, 0.4, and 1.0 W kg^−1^), data were fit to a linear model, and time point and dosage level were considered as covariates in determining significance.

## Results

### Assessment of cell proliferation in the presence of RF EMF

**Figure 2:**
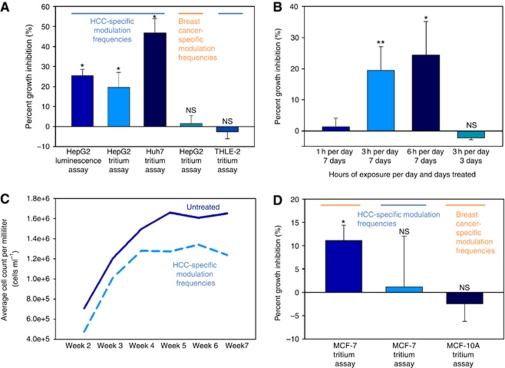
Cell proliferation assays of cell lines exposed to HCC-specific or breast cancer-specific modulation frequencies. (**A**) Cells were not split after initial seeding; medium was exchanged every 48 h. Experiments were performed with both equipment setups. Left to right columns: (1) HepG2 cells exposed to HCC-specific modulation frequencies with GI evaluated with a luminescence assay, 25.46±3.22% GI (*P*=0.0009). (2) HepG2 cells exposed to HCC-specific modulation frequencies with GI evaluated using tritium incorporation, 19.44±7.60% GI (*P*=0.00993). (3) Huh7 cells exposed to HCC-specific modulation frequencies, 47.73±7.14% GI (*P*=0.018). (4) HepG2 cells are not significantly inhibited when exposed to breast cancer-specific modulation frequencies, 1.49±3.99% GI (*P*=0.8815). (5) THLE-2 cells are not affected by HCC-specific modulation frequencies, −2.54±3.54% GI (*P*=0.6550). Values represent average percent GI (*n*=6)±%STERR. (**B**) Cell proliferation assays exposing cells for varying hours per day. Left to right: 1 h per day 1.36±2.77% (*P*=0.8508); 3 h per day 19.44±7.60% (*P*=0.0099); 6 h per day 24.46±10.75% (*P*=0.0301); 3 h per day for 3 days −2.12±0.66% (*P*=0.4067). Values represent average percent GI (*n*=6)±%STERR. (**C**) Cumulative decrease in cell counts over time when HepG2 cells are exposed to HCC-specific modulation frequencies. Samples were subcultured by volume every 7 days (1 : 20 split by volume). Average total cells mL^−1^ per week: week 2: 7.07 × 10^5^, 4.75 × 10^5^; week 3: 1.20 × 10^6^, 1.01 × 10^5^; week 4: 1.50 × 10^5^, 1.28 × 10^5^; week 5: 1.66 × 10^5^, 1.22 × 10^5^; week 6: 1.61 × 10^6^, 1.34 × 10^6^; week 7: 1.65 × 10^6^, 1.24 × 10^6^ for untreated and treated samples, respectively. For the duration of the 7-week experiment with time considered as a covariate: *P*=0.005751. (**D**) Left to right columns: (1) MCF-7 cells exposed to breast tumour-specific modulation frequencies, 11.08±3.30% GI (*P*=0.0230). (2) MCF-7 cells are not significantly inhibited when exposed to HCC-specific modulation frequencies, 1.49±3.99% (*P*=0.8815) GI, respectively. (3) MCF-10A cells are not affected by breast tumour-specific modulation frequencies, −2.46±3.75% GI (*P*=0.8579). Values represent average percent GI (*n*=6)±%STERR.

Cell proliferation assays were conducted after 7 days, that is, 21 h of exposure to amplitude-modulated RF EMF. Treatment with HCC-specific modulation frequencies (Supplementary Table 1) significantly reduced the proliferation of HepG2 and Huh7 cells using both the parallel plate capacitor and the transverse electromagnetic (TEM) setups (Figure 1). The observed growth-inhibitory effect on HepG2 cells was of the same magnitude when using a tritium incorporation assay and a bioluminescence assay based on ATP consumption (Figure 2A). Having shown similar results with two different assays, the remainder of the cell proliferation experiments were conducted with the more commonly used tritium incorporation assay. Cell proliferation of HepG2 and Huh7 cells exposed to HCC-specific modulation frequencies was significantly lower than the proliferation of cells exposed either to randomly chosen frequencies (Supplementary Table 2) or not exposed to RF EMF (Figure 2A, columns 1–3). When HepG2 cells were exposed for only 1 h daily, we did not observe any significant inhibition of cell proliferation (Figure 2B). Daily exposure for 6 h instead of 3 h resulted in the same level of cell-proliferation inhibition (Figure 2B). To determine when HCC-specific modulation frequencies begin to exert anti-proliferative effects on HepG2 cells, we assessed cell proliferation following 3 days (9 h) of exposure and did not find any significant difference between cells exposed to HCC-specific modulation frequencies and unexposed cells (Figure 2B).

Further, to determine whether the growth-inhibitory effect of HCC-specific modulation frequencies persists over time and results in a decrease in the total number of tumour cells, we counted the number of HepG2 cells following treatment with HCC-specific modulation frequencies and that of untreated HepG2 cells weekly for up to 7 weeks. Cells that were either exposed to HCC-specific modulation frequencies or not exposed were split weekly at the same ratio over a period of 7 weeks. As shown in Figure 2C, when compared with unexposed HepG2 cells, the number of HepG2 cells following exposure to HCC-specific modulation frequencies decreased steadily over 7 weeks, resulting in a cumulative loss of 1.71 × 10^6^ cells per ml at week 7.

The average specific absorption rate (SAR) for cells exposed in the parallel capacitor plate system is 0.03 W kg^−1^ (Supplementary Information). All initial experiments conducted with the TEM system were conducted at a SAR of 0.4 W kg^−1^. To determine the range of SARs within which significant GI was observed, additional cell proliferation experiments were performed at 0.05, 0.1 and 1.0 W kg^−1^. A significant anti-proliferative effect was observed at all SARs ranging from 0.05 to 1.0 W kg^−1^ (*P*=0.0354). All subsequent assays with the TEM system were conducted at an SAR of 0.4 W kg^−1^.

### Inhibition of cell proliferation is tumour and tissue specific

Our previous clinical observations revealed that patients with HCC had biofeedback responses to specific modulation frequencies that were different from those identified in patients with other types of cancer, such as breast cancer (Barbault *et al*, 2009). To experimentally assess the relevance of these findings on the proliferation of tumour cells, we determined the specificity of frequencies identified in patients with these two tumour types given the documented objective clinical responses that included one complete and one partial response in two patients with metastatic breast cancer (Barbault *et al*, 2009) and three partial and one near-complete responses in four patients with HCC (Costa *et al*, 2011). A total of 194 breast cancer-specific modulation frequencies ranging in the same modulation frequency band from 100 Hz to 21 kHz have been identified in patients with a diagnosis of breast cancer (Supplementary Table 3). In all 9 (4.6%) of the HCC-specific modulation frequencies are identical to breast cancer-specific modulation frequencies.

The two patients with metastatic breast cancer who had experienced an objective response to breast cancer-specific modulation frequencies had tumours that over-expressed oestrogen receptor (ER+) and progesterone receptor (PR+), but did not over-express ERBB2 (ERBB2−) (Barbault *et al*, 2009). We therefore chose the MCF-7 cell line as it represents the same tumour phenotype, that is, ER+, PR+, ERBB2−. Although the growth of MCF-10A breast cells was unaffected by exposure to breast cancer-specific modulation frequencies, exposure of MCF-7 breast cancer cells to breast cancer-specific modulation frequencies significantly inhibited cell proliferation (Figure 2D). However, exposure of HepG2 cells to the same breast cancer-specific modulation frequencies did not affect cell proliferation (Figure 2A). Similarly, the proliferation of MCF-7 cells was not affected by exposure to HCC-specific modulation frequencies (Figure 2D). Consequently, the observed anti-proliferative effect on HCC and breast cancer cells was observed only upon exposure to tumour-specific modulation frequencies previously identified in patients with a diagnosis of HCC and breast cancer, respectively, despite the fact that more than 57% of the modulation frequencies only differed by <1% (Supplementary Tables 1 and 3).

Having demonstrated that the anti-proliferative effect of amplitude-modulated frequencies was tumour specific, we sought to determine whether the HCC-specific modulation frequencies have an effect on the proliferation of THLE-2 normal hepatocytes. As shown in Figure 2A, exposure of THLE-2 cells to HCC-specific modulation frequencies did not have any measurable effect on cell proliferation. These findings provide strong support for the novel notion that a combination of narrowly defined, specific modulation frequencies identified in a group of patients with the same type of cancer is capable of inhibiting cell proliferation in a tumour- and tissue-specific fashion.

### Tumour-specific modulation frequencies and gene regulation

**Figure 3:**
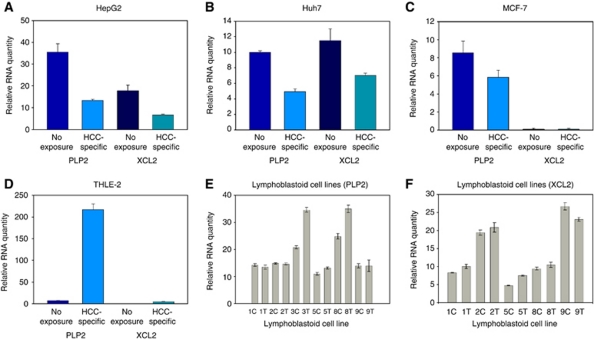
Expression of *XCL2* and *PLP2* receiving HCC-specific RF EMF compared with cells not receiving exposure. (**A**) HepG2: *PLP2* (35.46±3.85; 13.17±0.70) and *XCL2* (17.87±2.49; 6.52±0.48) (*P*=9.0371e-3 and *P*=0.0179, respectively). (**B**) Huh7: *PLP2* (10.02±0.19; 4.95±0.35) and *XCL2* (11.52±1.49; 7.02±0.29) (*P*=9.4981e-5 and *P*=0.0536, respectively). (**C**) MCF-7: *PLP2* (8.52±1.30; 5.84±0.77) and *XCL2* (levels not detectable). (**D**) THLE-2: *PLP2* (7.11±0.14; 216.89±13.18) and *XCL2* (0.03±0.01; 4.55±1.04) in THLE-2 cells exposed to HCC-specific modulation frequencies (*P*=5.5108e-4 and *P*=0.0221, respectively). (**E**) Expression levels of *PLP2* in lymphoblastoid cell lines (C=unexposed; T=HCC-specific exposure) (for all cell lines compiled *P*=0.418), LCL 3 expression was significant *P*=0.0021 as was LCL8 *P*=0.0159 (**F**) Expression levels of *XCL2* in lymphoblastoid cell lines (for all cell lines compiled (*P*=0.899), LCL 1 expression difference was significant *P*=0.0002. Values represent average relative RNA expression (*n*=4)±s.e.m. Levels were normalised to levels of GAPDH.

To study the mechanism by which tumour-specific modulation frequencies inhibit cell proliferation, we assessed the gene expression profile of HepG2 cells exposed to HCC-specific modulation frequencies using RNA-seq, as it provides a more comprehensive assessment of differential gene expression across a broader range of expression levels than microarray-based analysis (Wang *et al*, 2009). Overall, we did not observe statistically significant differences in transcript levels when comparing two HepG2 cultures exposed for 1 week, 3 h a day to HCC-specific modulation frequencies with two HepG2 cultures exposed to randomly chosen modulation frequencies (Supplementary Figure 1). However, we did observe a small number of genes with an absolute fold-change >1.5 and a minimum mean RPKM of 1.5 following exposure to HCC-specific modulation frequencies. Two genes with an absolute fold-change >1.8 appeared to be downregulated in HepG2 cells exposed to HCC-specific modulation frequencies, *PLP2* and *XCL2*, and were considered to be candidates worthy of further experiments. We validated the downregulation of *PLP2* and *XCL2* with quantitative PCR in both HepG2 as well as Huh7 cells exposed to HCC-specific modulation frequencies (Figures 3A and B). There was no significant downregulation of *PLP2* and *XCL2* in MCF-7 breast cancer cells (Figure 3C). Similarly, there was no downregulation of *PLP2* and *XCL2* in nonmalignant cells, that is, in THLE-2 normal hepatocytes (Figure 3D), or in lymphoblastoid cell lines from healthy individuals (Figures 3E and F). These findings support the novel notion that the demodulation effects of RF EMF amplitude-modulated at specific frequencies inhibit cell proliferation and affect the expression of several genes in a tumour- and tissue-specific fashion.

### Tumour-specific modulation frequencies and disruption of the mitotic spindle

There is evidence that the proliferation of several rodent and human cancer cell lines is arrested by exposure to sinusoidal electric fields of 100–200 V m^−1^ at a frequency of 100–300 kHz (Kirson *et al*, 2004). This approach has also shown efficacy in animal and human tumour models as well as promising results in the treatment of patients with cancer (Kirson *et al*, 2004; Kirson *et al*, 2007; Salzberg *et al*, 2008; Kirson *et al*, 2009). The anti-tumour effect of this therapeutic approach appears to be caused by disruption of the mitotic spindle mediated by interference of spindle tubulin orientation and induction of dielectrophoresis (Kirson *et al*, 2004; Kirson *et al*, 2007). In contrast to the sinusoidal signals (Kirson *et al*, 2004), the carrier frequency of the signal applied in our experiments is more than 100 times higher; the peak E-field amplitude of the carrier at 0.4 W kg^−1^ corresponds to approximately 35 V m^−1^ inside the cell medium when the signal is sinusoidally amplitude-modulated at specific frequencies with 85% modulation depth (Kirson *et al*, 2004).

**Figure 4:**
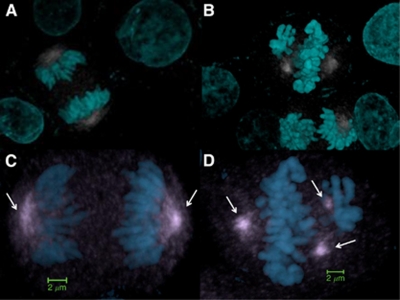
Mitotic spindle disruption in cells receiving HCC-specific RF EMF compared with cells not receiving exposure. (**A**) HepG2 efficiently assembles a bipolar mitotic spindle, allowing cells to pass through the mitotic assembly checkpoint and successfully progress from metaphase to anaphase, (**B**) >60% of dividing HepG2 cells exposed to HCC-specific modulation frequencies exhibit microtubule-associated anomalies, (**C**) high magnification of unexposed HepG2 cells in mitosis (**D**) high magnification of HepG2 cells exposed to HCC-specific modulation frequencies shows errors such as tripolar spindle formation (Cyan=DAPI; Gray=Microtubules; Arrows=mitotic spindle).

Despite these significant differences, confocal laser scanning microscopy revealed pronounced disruption of the mitotic spindle in more than 60% of HepG2 cells exposed for 1 week, 3 h per day to HCC-specific modulation frequencies whereas there was no disruption of the mitotic spindle in unexposed HepG2 cells (Figures 4A and B). Specifically, the observed cytoskeletal disruption in cells exposed to HCC-specific modulation frequencies was apparent in cells in mitosis, in which we saw centrosomal distortion and poor chromosomal separation at anaphase (Figure 4D). We found no evidence of karyotypic differences between HepG2 cells exposed to HCC-specific modulation frequencies and unexposed HepG2 cells.

## Discussion

By exposing HCC cells to 27.12 MHz RF EMF sinusoidally amplitude-modulated at specific frequencies, which were previously identified in patients with a diagnosis of HCC (Barbault *et al*, 2009) and result in therapeutic responses in patients with HCC (Costa *et al*, 2011), we demonstrate a robust and sustained anti-proliferative effect. This effect was seen within SARs ranging from 0.03 to 1.0 W kg^−1^, that is, within the range of exposure in humans receiving treatment administered intrabuccally (Barbault *et al*, 2009; Costa *et al*, 2011). HCC-specific modulation frequencies began to hinder cell proliferation after 7 days of exposure and the anti-proliferative effect increased over a 7-week period. The anti-proliferative effect HCC-specific modulation frequencies were observed only in HCC cells, but not in breast cancer cells or normal hepatocytes.

The specificity of modulation frequencies is exemplified by the fact that two sets of similar modulation frequencies (breast cancer-specific and randomly chosen) within the same range, that is, from 100 Hz to 21 kHz, did not affect the proliferation of HCC cells. Similarly, the proliferation of breast cancer cells was affected only by breast cancer-specific modulation frequencies, but neither by HCC-specific nor by randomly chosen modulation frequencies. The fact that >50% of the modulation frequencies from these three programs differed by <1%, provides strong experimental evidence that the biological effects are only mediated by a combination of narrowly defined, tumour-specific modulation frequencies.

The modulation-frequency specific laboratory findings are consistent with the clinical observation of a complete response in a patient with breast cancer metastatis to the adrenal gland and the bone while a primary malignancy of the uterus continued to grow (Barbault *et al*, 2009). This suggests that a combination of precise tumour-specific modulation frequencies is needed to block cancer growth *in vitro* and in patients with a diagnosis of cancer. The clinical results reported by Barbault *et al*, (2009) and Costa *et al*, (2011) as well as laboratory evidence included in this report provide support for the novel and transformational concept that the growth of human tumours arising from the same primary tissue may be effectively blocked by identical modulation frequencies. While receiving treatment with HCC-specific modulation frequencies, one black and three white patients with advanced carcinoma had partial responses (Costa *et al*, 2011). Furthermore, proliferation of the Huh7 HCC cell line, which is derived from a Japanese patient's tumour (Nakabayashi *et al*, 1982), exhibited the most pronounced response to HCC-specific modulation frequencies (Figure 2A). This indicates that the frequency signature and biological effects of HCC-specific modulation frequencies are likely independent of ethnic status.

There is no known biophysical mechanism accounting for the effect observed in these experiments; however, other modulation-frequency dependent effects have been observed in biological systems at similarly low exposure levels. Documented effects have occurred in cellular processes controlling cell growth, proliferation, and differentiation (Blackman, 2009). Further, modulation of the signal appears to be a critical factor in the response of biological systems to electromagnetic fields (Blackman, 2009). The amount of electromagnetic energy delivered is far too low to break chemical bonds or cause thermal effects, necessitating alternative mechanistic explanations for observed biological outcomes. Several theories have been put forth to explain biological responses to electromagnetic fields. Some reports have shown that low levels of electromagnetic fields can alter gene expression and subsequent protein synthesis by interaction of the electromagnetic field with specific DNA sequences within the promoter region of genes (Blank and Goodman, 2008; Blank and Goodman, 2009). Such changes have been demonstrated in the family of ‘heat shock’ proteins that function in the cell stress response (Blank and Goodman, 2009).

To thoroughly interrogate gene expression changes in cells exhibiting decreased cell proliferation, we used high-throughput sequencing technologies to sequence the cells' cDNA, a technique that has become invaluable in the study of cancer (Maher *et al*, 2009). Tumour cell GI was associated with downregulation of *PLP2* and *XCL2* as well as with disruption of the mitotic spindle. *PLP2* encodes an integral membrane protein that localises to the endoplasmic reticulum in epithelial cells. The encoded protein can multimerise and may function as an ion channel (Breitwieser *et al*, 1997). *PLP2* enhances chemotaxis of human osteogenic sarcoma cells (Lee *et al*, 2004) and *PLP2* downregulation is associated with decreased metastasis in a mouse model of cancer (Sonoda *et al*, 2010). *XCL2* encodes for a protein that enhances chemotactic activity for lymphocytes and downregulation of *XCL2* has been shown to be associated with good prognosis in patients with breast cancer (Teschendorff *et al*, 2007; Teschendorff and Caldas, 2008). The pronounced disruption of the mitotic spindle seen in the majority of HepG2 cells exposed to HCC-specific modulation frequencies undergoing mitosis is not associated with karyotypic changes, but may be a major determinant of the anti-tumour effects of HCC-specific modulation frequencies accounting for the therapeutic responses seen in patients receiving the same modulation frequencies (Costa *et al*, 2011).

Exposure of HCC cells to the same RF EMF modulated at slightly different modulation frequencies did not result in changes in gene expression, which demonstrates that inhibition of cell proliferation is associated with changes in gene expression levels.

In conclusion, we show that very low levels of 27.12 MHz radiofrequency electromagnetic fields, which are comparable to the levels administered to patients, inhibit tumour cell growth when modulated at specific frequencies. The exciting findings presented in this report suggest that the anti-proliferative effect of modulation frequencies is both tumour- and tissue-specific, and is mediated by changes in gene expression as well as disruption of the mitotic spindle. These findings uncover a new alley to control tumour growth and may have broad implications for the treatment of cancer.
