# Supplementary material for: Cancer cell proliferation is inhibited by specific modulation frequencies
Source: Br J Cancer. 2011 Dec 1;106(2):307–13. doi: 10.1038/bjc.2011.523 (PMC3261663; doi:10.1038/bjc.2011.523)
Supplement: Supplementary Figure 1 [file bjc2011523x1.pdf]

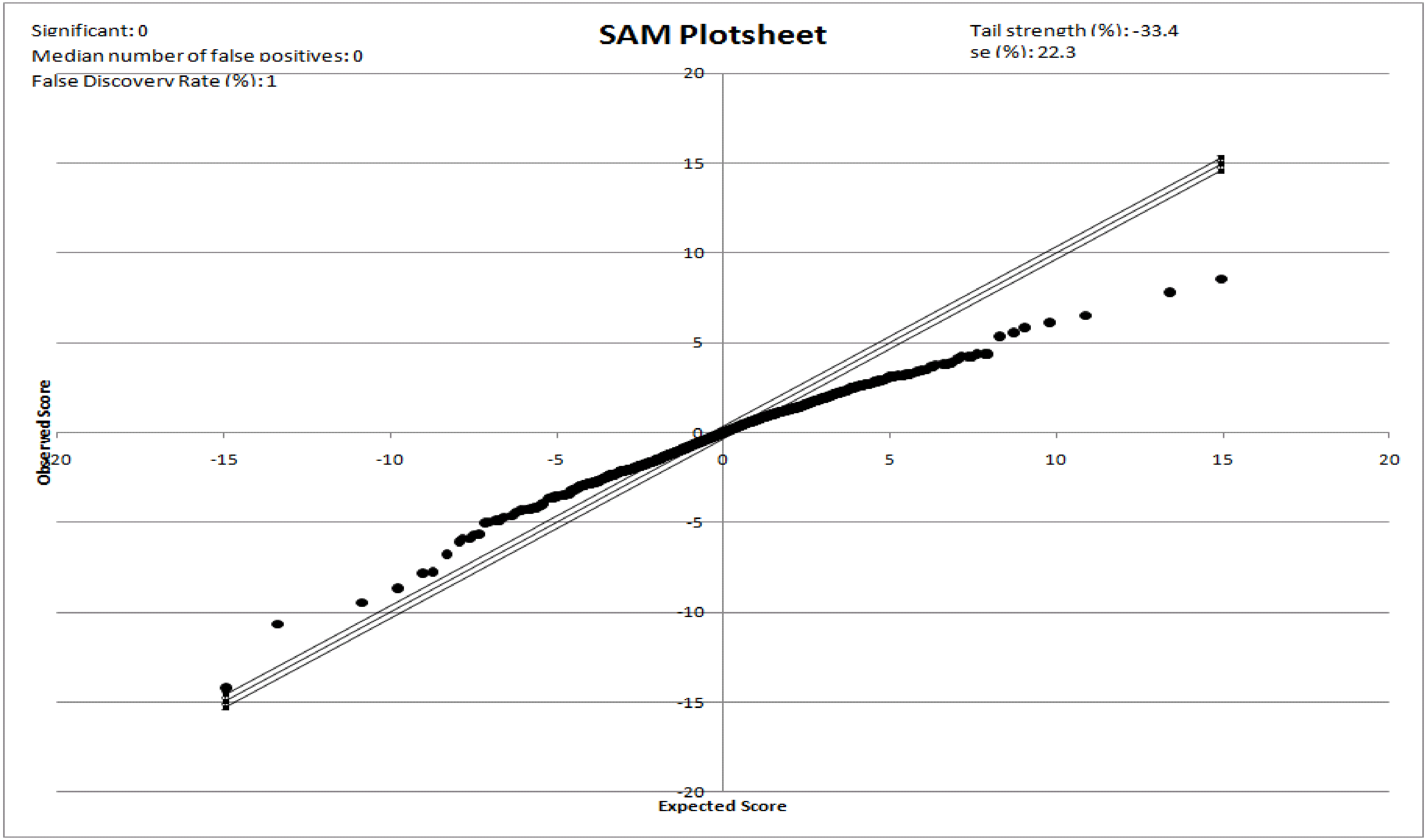
Significant: 0
Median number of false positives: 0
False Discovery Rate (%): 1
SAM Plotsheet
Tail strength (%): -33.4
se (%): 22.3
20
15
10
5
0
-5
-10
-15
-20
-20
-15
-10
-5
0
5
10
15
20
Observed Score
Expected Score
