# Supplementary figures and images for: Cancer cell proliferation is inhibited by specific modulation frequencies
Source: Br J Cancer. 2011 Dec 1;106(2):307–13. doi: 10.1038/bjc.2011.523 (PMC3261663; doi:10.1038/bjc.2011.523)

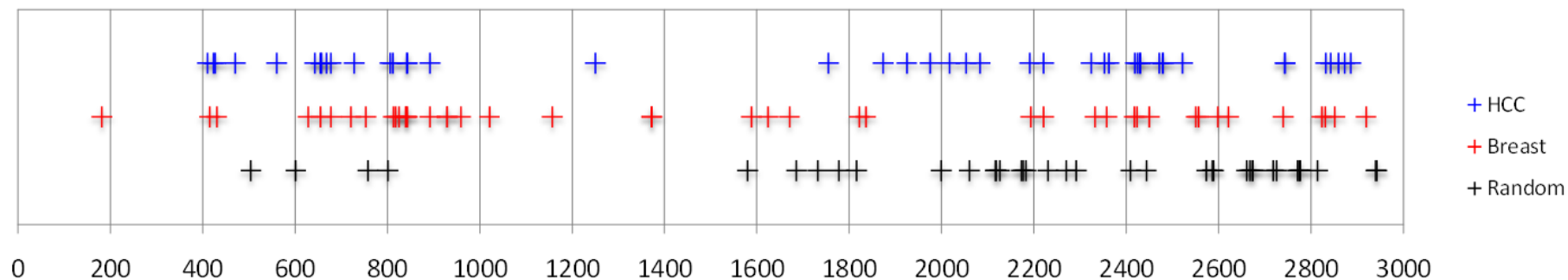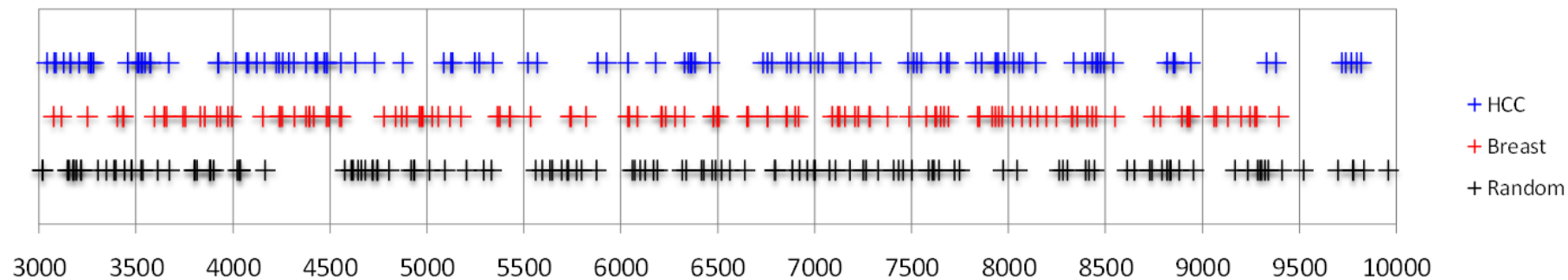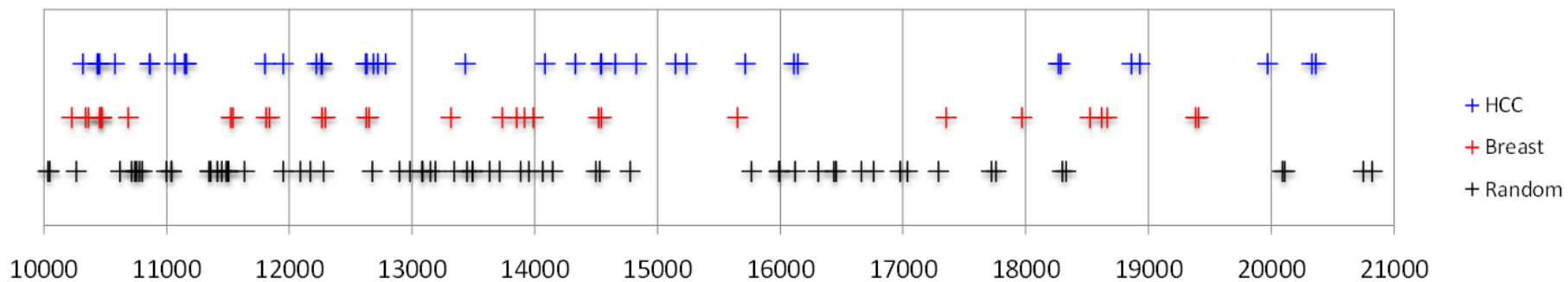

Supplement: Supplementary Figure 2 [file bjc2011523x2.pdf]
