# Supplementary material for: Cancer cell proliferation is inhibited by specific modulation frequencies
Source: Br J Cancer. 2011 Dec 1;106(2):307–13. doi: 10.1038/bjc.2011.523 (PMC3261663; doi:10.1038/bjc.2011.523)
Supplement: Supplementary Information [file bjc2011523x3.doc]

**Supplementary notes**

**1 Design and construction of *in vitro* exposure devices**

1.1 Exposure system connected to the same devices as those use for clinical studies. An apparatus for cell irradiation was designed and built to expose cell cultures to the same radiofrequency (RF) signal as the one used for treatment of patients. Briefly, the design of four identical exposure chambers is based on a parallel plate capacitor (14 x 21 cm) arrangement excited by devices identical to the ones used for clinical studies (the outer two plates on the device ground and the center plate on the active port to establish symmetry) (Figure 1A). The outer brass plates are connected to the outer conductor of the coaxial cable, whereas the center brass plate is connected to the inner conductor of the coaxial cable. Connections are made near the middle of the narrow edges of the plates (Figure 1A).

One set of devices is programmed with the same HCC-specific or breast cancer-specific frequencies as those used for patient treatment (Suppl. Tables 1 and 3, Suppl. Figure 2). Another set of devices is programmed with randomly-chosen frequencies selected in the same band as the HCC-specific frequencies, i.e. from 100 Hz to 21 kHz (Suppl. Table 2, Suppl. Figure 2). Additional control devices consisted of the same devices, which were not switched on. Temperature of the culture medium was measured before and after treatment. There was no measurable difference in the temperature of the media during and following exposure to amplitude-modulated frequencies in the parallel plate capacitor. The averaged induced SAR at the monolayer was assessed to be 0.034 W/kg (uncertainty of 40%) and a standard deviation of 155% (highest cell exposures at the edge of the dish and smallest exposures in the center).

1.2 sXc27 TEM System. An optimized system for well controlled exposure of cell cultures to 27.12 MHz radiofrequency (RF) electromagnetic fields was developed, manufactured, and characterized by the Foundation for Research in Information Technologies in Society (IT'IS Foundation, Zurich, Switzerland). The system is based on two identical transverse electromagnetic (TEM) cells (IFI CC110, Instruments for Industry Inc., Ronkonkoma N.Y.) that can be loaded with 2 x 6 tissue culture dishes (35 mm, FALCON, monolayer cells) (Figure 1B). The dish holder is made out of polyoxymethylene (POM, relative permittivity = 3.5 (50%) conductivity = 0.0001 (50%) at the 27 MHz and 37°C). The design is an adaptation of the system described by Nikoloski et al.(2005). The propagation vector is normal to the bottom of the tissue culture dish allowing homogenous exposure of cell monolayers and cells in suspension. A constant airflow is forced through the system by fans with a common inlet ensuring the same environmental conditions as inside the incubator. The controlling and monitoring unit generates the exposure signal via computer-controlled signal generators and monitors the exposure levels using power sensors at the output of the TEM cells. It also monitors temperature and the functioning of the fans every 5s. All measured and control parameters are stored in a log file. Since the system consists of two identical chambers, it allows blinded exposure schemes with active and inactive RF-modulations, i.e. the computer randomly selects the active versus inactive signals, the information of which is only accessible via the log file.

The specific absorption rate (SAR) distribution within the cell culture dish was characterized with the electromagnetic and thermal simulation platform SEMCAD X V14 that was developed by the IT’IS Foundation and SPEAG (Zurich, Switzerland) (Figure 1C). The results of the simulations were validated by measurements using temperature probes T1V3lab (SPEAG, Zurich).

The efficiency of the exposure system for the cell monolayer is 10.7 mW/kg per Watt of forward power for the MCF-7 medium (relative permittivity = 75 (2.5%), conductivity = 1.66 (5%) at the 27 MHz and 37°C) and 10.2 mW/kg for the HepG2 medium (relative permittivity = 74.3 (2.5%), conductivity = 1.74 (5%) at the 27 MHz and 37°C). The variation of averaged SAR between the six tissue culture dishes is less than +/-8% (Figure 1C). The non-uniformity of the cell monolayer exposure is 19% (SD, k=1) for all dishes. The maximum possible temperature increase inside the cell medium due to RF exposure is < 0.1°C per W/kg averaged exposure. The combined relative uncertainty for the whole dosimetric assessment is 23%, and SAR variability between experiments is ≤ 6%. Temperature difference in the sXc27 system between exposed and unexposed was less than 0.04°C at 0.4 W/kg.

Reference List

1. Nikoloski N, Frohlich J, Samaras T, Schuderer J, Kuster N (2005) Reevaluation and improved design of the TEM cell in vitro exposure unit for replication studies. *Bioelectromagnetics* **26**(3):215-224.

**Supplementary Figure 1**

**RNA-Seq assay of HepG2 cells exposed to HCC-specific vs. randomly chosen modulation frequencies.**

**Supplementary Figure 2**

**Graphical representation of the HCC-specific, randomly-chosen and breast cancer-specific frequencies.** Frequencies are located in the 100 Hz to 21 kHz band.
